# From automation to symbiosis in Industry 5.0 manufacturing: the human-centered AI adoption maturity cube

**DOI:** 10.3389/frai.2026.1913676

**Published:** 2026-08-21

**Authors:** Hugh Jiliang Liu

**Affiliations:** 1 International Business Unit, Business and Law Cluster, Hanze University of Applied Sciences, Groningen, Netherlands; 2 Department of Psychology, Faculty of Behavioral and Social Sciences, University of Groningen, Groningen, Netherlands

**Keywords:** AI Governance, empowerment, human-AI symbiosis, human-centered AI, Industry 5.0, manufacturing work, maturity model

## Abstract

Manufacturing firms are increasingly adopting AI to improve inspection, maintenance, scheduling, robotics, digital twins, knowledge management, and supply chain coordination. However, these applications do not automatically create human-centered progress. Without deliberate organizational maturity, AI may strengthen technical performance while weakening worker agency, trust, inclusion, and accountability. This article proposes the Human-Centered AI Adoption Maturity Cube (HAIAM Cube) as a conceptual framework intended to support structured reflection on AI adoption in Industry 5.0 manufacturing. Drawing on literature on digital transformation, Industry 5.0, human-centered AI, trustworthy AI, work design, empowerment, inclusivity, and maturity models, the article argues that mature AI adoption depends on the alignment of three interdependent capabilities: AI and data capability, human work capability, and governance capability. The cube conceptually links these dimensions to four desired outcomes for future manufacturing jobs: augmentation, empowerment, inclusivity, and human-AI symbiosis. Rather than treating maturity as a linear sequence, the framework positions organizations according to different maturity configurations, such as fragmented pilots, technical acceleration, participatory but fragile adoption, governed but disconnected adoption, and symbiotic maturity. The article offers a modest scaffold for managers, engineers, worker representatives, and researchers seeking to align AI adoption with responsible and human-centered industrial renewal, while providing a conceptual basis for future assessment development and empirical validation.

## 1 Introduction

Manufacturing is entering a period in which the adoption of artificial intelligence (AI) can no longer be treated as a peripheral technology project. Digital transformation reshapes value creation, organizational structures, and routines rather than simply adding tools (Vial, 2019). In manufacturing, this shift is intensified by global competition, fragile supply networks, decarbonization pressures, demographic change, and persistent skills shortages. At the same time, AI is moving beyond isolated pilots into inspection, maintenance, scheduling, robotics, digital twins, knowledge management, and supply chain coordination (Passalacqua et al., 2025; Soori et al., 2023). This movement creates

urgency because decisions about data, governance, job design, and workforce participation may determine whether AI strengthens manufacturing capability or narrows work into monitoring tasks and diminishes human agency. Since these outcomes depend less on AI itself than on the conditions under which it is designed, implemented, and governed, AI adoption should be understood as a question of maturity. The central issue is therefore not only whether manufacturers can adopt AI, but whether they can develop and sustain the capabilities required for mature AI adoption.

In manufacturing, AI commonly refers to computational systems that infer patterns from data to support prediction, recommendation, classification, or action (OECD, 2024). This definition matters because AI rarely remains neutral once it enters production. It redistributes attention, expertise, accountability, and voice by changing what workers monitor, what managers measure, and which forms of judgment become visible or invisible (Parker and Grote, 2022). A vision system may shift inspectors from direct defect detection to exception handling and model interpretation. A predictive maintenance model may reshape relationships between technicians and engineers. A planning algorithm may influence how supervisors negotiate speed, safety, flexibility, and resources (Maddikunta et al., 2022; Wuest et al., 2016). These changes can improve quality, resilience, customization, and organizational learning when embedded in human-centered Industry 5.0 principles (Breque et al., 2021; Xu et al., 2021). However, technical performance alone does not ensure that AI will be trustworthy, accountable, or usable. Without deliberate maturity, adoption may remain technically impressive but socially fragile, especially when workers have limited influence over systems that reshape task boundaries, decision rights, and career mobility (Parker and Grote, 2022; Vial, 2019). This concern aligns with research showing that automation can both substitute and complement work, that human-AI symbiosis depends on combining computational capability with human contextual judgment, and that automation and augmentation are often intertwined rather than separable pathways (Jarrahi, 2018; Raisch and Krakowski, 2021).

The promise of AI adoption is substantial, but its risks are equally clear. When adoption is framed mainly as automation, organizations may develop sophisticated systems that reduce discretion, weaken trust, or exclude employees who are not digitally fluent. When adoption is framed mainly as participation, organizations may underinvest in data quality, analytical capability, and responsible governance. Industry 5.0 helps hold these concerns together. It complements Industry 4.0 by placing human-centeredness, sustainability, and resilience at the center of technological progress (Breque et al., 2021). Rather than rejecting digital manufacturing, it asks whether digital manufacturing serves broader social and organizational purposes. Xu et al. (2021) describe this transition as a shift from technology-driven industrialization toward value-driven industrialization. This distinction is crucial for AI because the same algorithm may either narrow work into monitoring and exception handling or expand it through better insight, safer collaboration, and new expertise. The difference lies not in the model alone, but in the work system in which it is embedded. In this context, this article proposes a conceptual framework intended to organize structured reflection and generate propositions for future testing, rather than to claim demonstrated assessment accuracy or organizational effects.

## 2 Conceptual foundations

This article proposes the Human-Centered AI Adoption Maturity (HAIAM) Cube as a conceptual framework for supporting structured reflection on AI adoption in Industry 5.0 manufacturing. A maturity model represents the development of organizational capabilities through staged or multidimensional structures that support diagnosis, learning, and improvement (Becker et al., 2009). The HAIAM Cube is oriented toward four desired work-system outcomes central to future manufacturing work: augmentation, empowerment, inclusivity, and human-AI symbiosis. It is not presented as an empirically validated diagnostic instrument or a definitive solution, but as a theoretically grounded scaffold that organizations can adapt, test, and refine across manufacturing contexts.

Augmentation refers to the use of AI to extend human perception, reasoning, coordination, or action while preserving meaningful human contribution. Automation may substitute for some tasks while complementing others (Autor, 2015), and AI can automate parts of a process while reshaping the human work that remains (Raisch and Krakowski, 2021). For example, predictive maintenance may automate anomaly detection while helping technicians interpret degradation patterns and prioritize interventions. The key issue is therefore not simply whether a task is automated, but whether the broader work system preserves, develops, and productively uses human judgment.

Empowerment refers to a motivational state expressed through meaning, competence, self-determination, and impact (Spreitzer, 1995). In AI adoption, empowerment goes beyond training hours or dashboard access. Employees may know how to use an AI system but still lack empowerment if its assumptions are hidden, its recommendations are difficult to question, or new skills are not recognized. In AI-supported manufacturing, empowerment means that employees have the capability, confidence, and authority to understand, use, challenge, and shape AI-supported work. This view aligns with self-determination theory, which links motivation and wellbeing to autonomy, competence, and relatedness (Ryan and Deci, 2000), and with work design research showing that digital technologies shape autonomy, skill use, feedback, and participation (Parker and Grote, 2022).

Inclusivity refers to being valued as a member of a work group while being able to express one's uniqueness (Shore et al., 2011). In Industry 5.0 manufacturing, inclusivity means that AI adoption should be accessible and meaningful for workers with diverse abilities, ages, languages, educational backgrounds, roles, and levels of digital experience. It also requires treating shop-floor knowledge as design intelligence, not merely post-implementation feedback. Participatory design emphasizes that people affected by technology should help shape its design and use (Muller and Kuhn, 1993), while Suchman (2007) shows that technologies are enacted within situated practices rather than imposed on passive users. An inclusive approach therefore expands who can understand, question, adapt, and improve AI systems.

Human-AI symbiosis refers to a reciprocal work arrangement in which people and AI systems contribute complementary strengths to decision-making under uncertainty (Jarrahi, 2018). Building on Licklider's (1960) account of human-computer symbiosis, contemporary research emphasizes the combination of computational capability and human contextual judgment (Jarrahi, 2018). Humans contribute practical experience, ethical reasoning, social understanding, and purpose, while AI contributes scale, speed, pattern recognition, and consistency. In manufacturing, this complementarity matters because operational decisions often combine sensor data, production constraints, tacit knowledge, safety concerns, and customer requirements. Human-AI symbiosis is therefore more than usability. It requires an evolving relationship in which human practices, organizational routines, and AI models are continually adjusted through use, feedback, and learning.

Together, these four concepts are situated within the broader literature on human-centered and trustworthy AI. Human-centered AI scholarship proposes that high levels of automation can coexist with high levels of human control, rather than requiring reduced human agency (Shneiderman, 2020). Trustworthy AI emphasizes human agency, technical robustness, privacy, transparency, fairness, societal wellbeing, and accountability (Thiebes et al., 2021). For manufacturing, these perspectives suggest that AI maturity should be evaluated not only by technical performance but also by whether AI adoption is responsible, human-centered, and capable of improving the quality of work.

## 3 The human-centered AI adoption maturity (HAIAM) cube

**Figure 1:**
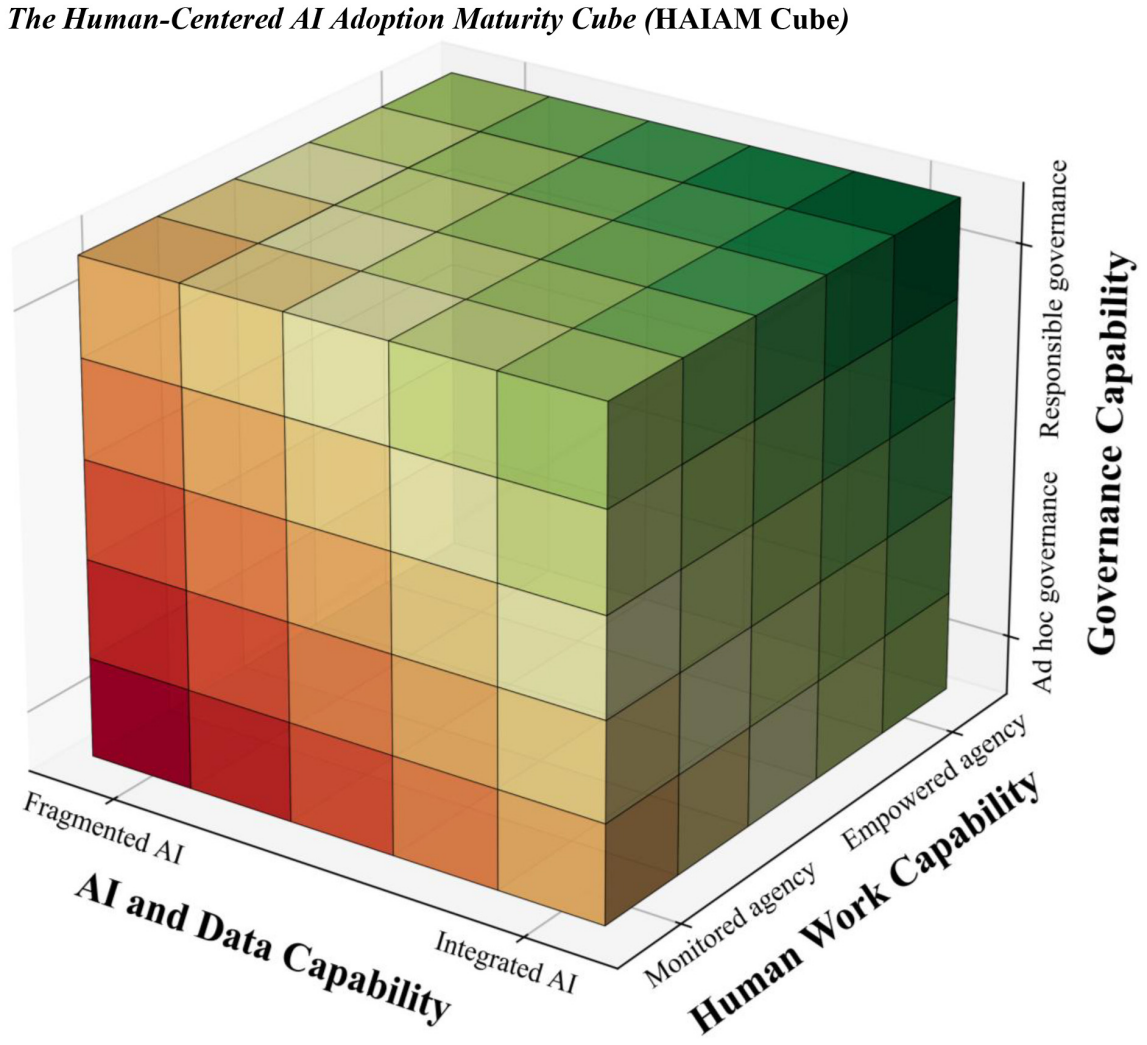
The human-centered AI adoption maturity cube (HAIAM cube).

The HAIAM Cube is presented in Figure 1 as a cube because AI maturity cannot be adequately represented as a single linear progression. A factory may possess advanced algorithms while still offering limited opportunities for worker voice. Another may have committed leaders and engaged employees but continue to rely on fragmented data infrastructure. A third may articulate careful ethical principles but lack mechanisms for translating those principles into everyday decisions about maintenance, inspection, or scheduling. The cube therefore conceptualizes maturity as the alignment of three interdependent dimensions, namely (1) AI and data capability; (2) human work capability; and (3) governance capability. The lower-maturity region represents less mature adoption, characterized by fragmented, opaque, and reactive practices. By contrast, the higher-maturity region represents more mature adoption, in which AI-related practices are reliable, participatory, and continuously improved. The color gradient is supplementary; maturity is determined by position along the three labeled axes rather than by color alone.

*AI and data capability* refers to an organization's ability to collect, integrate, validate, monitor, and improve production data and AI models, while providing appropriate explanations of model outputs in production. This dimension is central to smart manufacturing because AI depends on reliable data flows, interoperable systems, and learning mechanisms that connect digital models with operational realities (Kusiak, 2018; Tao et al., 2018). At lower levels of maturity, data are often dispersed across machines, spreadsheets, vendor systems, and informal records. Models may be developed for narrow pilots without clear responsibility for data quality, model drift, bias, explainability, or long-term maintenance. These weaknesses matter because concept drift can reduce model performance over time (Gama et al., 2014), while opaque models can make it difficult for users to understand or contest AI-supported recommendations (Guidotti et al., 2018; Rai, 2020). At higher levels of maturity, data are treated as a shared production resource. The organization has clear data ownership, interoperable systems, validation routines, model monitoring, and mechanisms for explaining model outputs in relation to production practice. Intermediate levels represent partial progress, where some systems may be connected and governed, but practices remain uneven across departments, sites, or use cases. This dimension does not imply that every manufacturer needs the most advanced algorithm. Instead, it suggests that AI cannot support mature work if its data foundation is unreliable, hidden, or disconnected from the conditions of production.

*Human work capability* refers to an organization's ability to design AI-supported jobs that preserve agency, develop skills, and enable meaningful participation. This dimension draws on sociotechnical thinking, which emphasizes that technical systems and work systems should be designed together rather than optimized separately (Clegg, 2000). It also reflects work design research showing that digital technologies can affect autonomy, skill use, feedback, participation, workload, and performance monitoring (Parker and Grote, 2022). At lower levels of maturity, AI is introduced as a tool that workers are expected to accept after technical decisions have already been made. Training focuses mainly on compliance rather than understanding, and supervisors may receive algorithmic output without guidance on when to trust, question, or override them. Such conditions can increase algorithmic control and weaken worker voice when people have limited opportunity to influence the systems that organize their work (Kellogg et al., 2020). At higher levels of maturity, workers participate early in design, testing, and evaluation. Job roles are redesigned so that people can use AI to diagnose problems, learn from exceptions, and improve processes. New skills are recognized within career pathways, and participation is treated as a production capability rather than a temporary change-management activity. Intermediate levels indicate a transitional condition in which workers may be consulted or trained, but their knowledge is not yet systematically embedded in AI design, evaluation, and continuous improvement.

*Governance capability* refers to an organization's ability to govern AI responsibly across risks, accountabilities, stakeholders, and life cycles. This dimension is necessary because AI adoption maturity depends not only on technical performance, but also on whether systems are transparent, safe, accountable, and aligned with human purposes. As noted earlier, human-centered AI should combine high levels of automation with high levels of human control (Shneiderman, 2020). Similarly, trustworthy AI emphasizes principles such as reliability, safety, transparency, and accountability (Jobin et al., 2019; Thiebes et al., 2021). However, principles alone are not sufficient unless organizations translate them into practical governance routines, responsibilities, and review mechanisms (Mittelstadt, 2019). At lower levels of maturity, governance is informal and reactive. Responsibility for AI decisions is unclear, risk assessment is inconsistent, and incidents may be treated as isolated technical problems. At higher levels of maturity, organizations maintain clear accountability, impact assessment, incident review, supplier transparency, worker consultation, and continuous learning. Intermediate levels reflect developing governance, in which policies or ethical principles may exist, but their application remains inconsistent across AI use cases. This dimension is especially important in manufacturing because AI often crosses organizational boundaries through vendors, machine builders, software platforms, customers, and regulators.

Although several practices span the AI-and-data and governance dimensions, the primary objects of diagnosis differ. AI and data capability focuses on the technical and operational management of data and model assets, including data quality, system interoperability, model validation, drift monitoring, bias testing, explainability techniques, and ongoing maintenance. Governance capability, by contrast, concerns organizational authority, oversight, and control, including decision rights, accountability arrangements, risk acceptance, documentation requirements, independent review, escalation procedures, incident response, and mechanisms for remedy. Explainability, bias management, validation, and data ownership should therefore be understood as boundary-spanning activities. Technical teams are responsible for implementing and maintaining the relevant tests, interfaces, and data-stewardship practices, whereas governance structures establish standards, assign accountable owners, evaluate evidence, and authorize corrective action. When applying the HAIAM Cube, a maturity gap should be located primarily according to whether it reflects a deficiency in technical execution or in organizational authorization and accountability. Where these functions are inseparable, the gap should be cross-referenced under both dimensions.

To make low, medium, and high maturity more concrete, without implying a validated scale, Table 1 provides concise, context-sensitive indicators. The indicators describe observable practices that organizations could discuss and document. They are not numerical thresholds and require adaptation to sector, firm size, use case, risk level, and institutional context.

**Table 1:** Illustrative indicators for low, medium, and high maturity across the three HAIAM dimensions.

Level	Low maturity	Medium maturity	High maturity
Dimension			
AI and data capability	Siloed or incomplete data; one-off pilots; unclear data/model ownership; *ad hoc* validation, explainability, and maintenance.	Partial interoperability; ownership and validation documented for some use cases; periodic monitoring; explainability and maintenance remain uneven across sites or systems.	Interoperable, quality-controlled data pipelines; lifecycle ownership; continuous validation and drift/bias monitoring; user-appropriate explanations; documented feedback and maintenance loops.
Human work capability	Technical decisions precede worker involvement; compliance-focused training; limited discretion, challenge, or override; skill changes and accessibility needs are weakly recognized.	Workers are consulted during testing; role-specific training and some escalation routes exist; participation, discretion, and job redesign vary by use case.	Early and continuing co-design; jobs support agency, learning, and complementary task allocation; clear challenge/override rights; inclusive access; new expertise is recognized in career pathways.
Governance capability	Informal, reactive troubleshooting; unclear accountability; no routine impact or risk review; incidents are handled locally with limited documentation.	Policies and responsible roles exist; periodic risk reviews and some supplier/incident documentation occur; implementation and worker representation remain inconsistent.	Documented accountability and risk tolerances; cross-functional lifecycle review; supplier transparency; worker representation; incident learning, remedy, auditability, and continuous improvement.

The indicators are illustrative and non-exhaustive. They do not constitute a validated scale, universal checklist, or claim that all practices must advance at the same rate.

The three dimensions are enabling conditions rather than one-to-one predictors of the four desired outcomes. Their contribution is joint and configurational: reliable AI and data can extend perception and make system behavior inspectable; human work capability converts technical potential into agency, learning, and meaningful participation; and governance capability defines legitimate purposes, decision rights, safeguards, review, and remedy. Table 2 makes these proposed pathways explicit. The pathways are conceptual propositions, not demonstrated causal effects, and future research needs to test their relative importance, interaction, and boundary conditions.

**Table 2:** Conceptual pathways from the three maturity dimensions to the four desired outcomes.

Outcomes	Augmentation	Empowerment	Inclusivity	Human-AI symbiosis
Dimensions			
AI and data capability	Reliable, context-relevant outputs extend perception, prediction, and decision quality.	Explainability and feedback enable understanding, challenge, and learning.	Accessible interfaces and bias monitoring reduce uneven access and effects.	Interoperable data and feedback loops support reciprocal adaptation of models and practice.
Human work capability	Complementary task allocation and job redesign combine human and AI strengths.	Participation, discretion, skill development, and override rights strengthen agency.	Co-design and accessible learning integrate diverse workers and situated expertise.	Continuing role calibration and mutual learning develop complementary collaboration.
Governance capability	Validation and risk review constrain unsafe, unreliable, or inappropriate use.	Decision rights, escalation routes, and accountability make influence contestable.	Representation, impact review, accessibility duties, and remedy address exclusion.	Lifecycle oversight and incident learning stabilize responsible, adaptive collaboration.

Mature adoption is represented by the joint configuration of the three dimensions, with the strongest potential for the desired outcomes expected when all three reach relatively high and mutually supportive levels. The matrix identifies the primary conceptual contribution of each dimension rather than exclusive or empirically established pathways.

## 4 Maturity positions in the HAIAM cube

**Figure 2:**
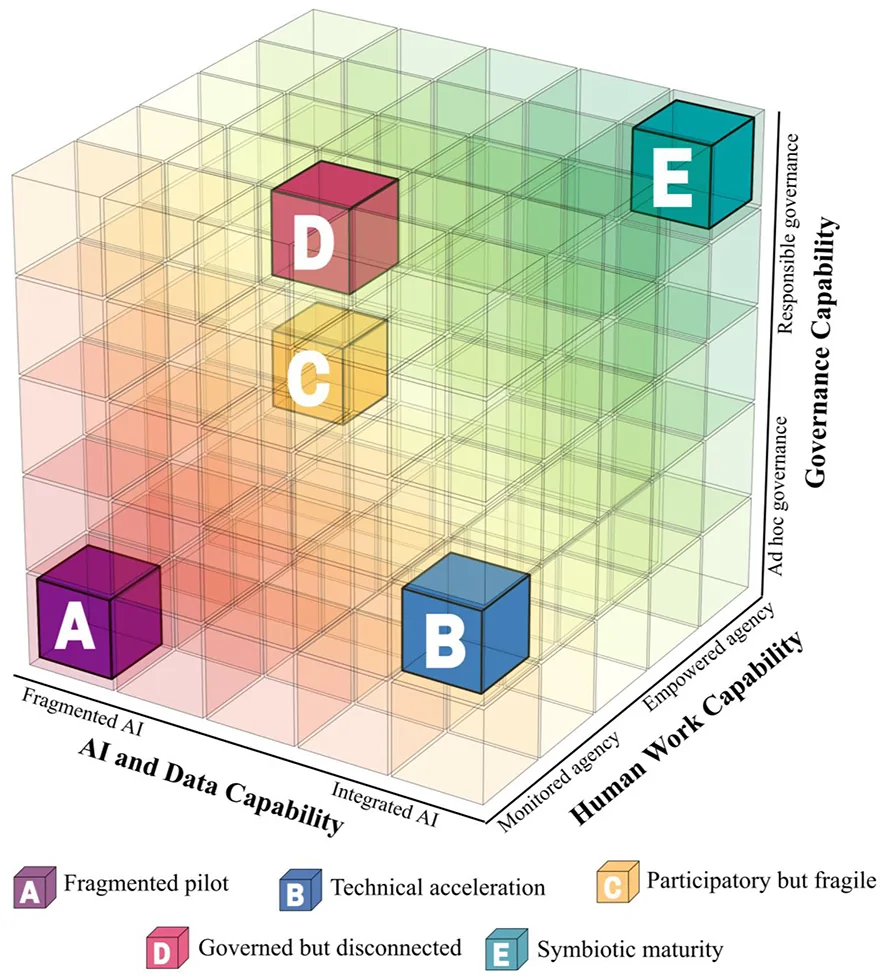
Illustrative maturity positions in the HAIAM Cube across the three maturity dimensions. “AI and data capability” ranges from fragmented pilots and siloed data to interoperable, validated, monitored, and explainable AI; “human work capability” ranges from monitored agency with post-implementation consultation to empowered agency with early and continuing participation; “governance capability” ranges from *ad hoc*, reactive oversight to documented, lifecycle-based responsible governance.

The HAIAM Cube should be read as a positioning framework rather than a linear stage model (see Figure 2). AI adoption maturity depends on an organization's joint position across three dimensions. A manufacturer may therefore be mature in one dimension while remaining less mature in another. This is why the cube can support structured reflection. It helps organizations characterize their current configuration, identify imbalance, and decide where improvement is most needed. The following are five illustrative examples of positions rather than exhaustive categories, summarized in Table 3.

**Table 3:** Illustrative maturity positions in the HAIAM cube.

Position	AI and data capability	Human work capability	Governance capability	Main improvement task
(A) Fragmented pilot	Low	Low	Low	Treat AI as sociotechnical change
(B) Technical acceleration	High	Low	Low to medium	Redesign work and clarify oversight
(C) Participatory but fragile	Low	High	Low to medium	Strengthen data and model foundations
(D) Governed but disconnected	Medium	Low to medium	High	Embed governance in daily work
(E) Symbiotic maturity	High	High	High	Sustain responsible adaptation

The five positions are illustrative configurations, not exhaustive categories, validated stages, or a required sequence of development.

A fragmented-pilot position occurs when all three dimensions remain weak. For example, a factory may test computer vision on one production line while data remain fragmented, workers are consulted only after implementation, and governance is limited to informal troubleshooting. This position is not necessarily a failure, since experimentation can yield useful learning. However, it is fragile because the pilot is treated mainly as technical proof rather than as a sociotechnical change.

A technical-acceleration position occurs when AI and data capability improve faster than human work capability and governance capability. For example, a predictive maintenance model may produce accurate anomaly scores, but technicians may not understand the model's assumptions or have authority to question recommendations. This position may create efficiency gains, yet it can also intensify monitoring and reduce agency if human roles are poorly designed.

A participatory-but-fragile position occurs when leaders and workers support AI adoption, but the data foundation remains unreliable. For example, operators may actively contribute knowledge about machine sounds, material variation, and recurring faults, yet the organization may lack interoperable systems, validation routines, or model monitoring. This position shows that participation alone is insufficient without dependable technical infrastructure.

A governed-but-disconnected position occurs when organizations have ethical principles, risk policies, or supplier requirements, but these are not yet translated into everyday decisions about maintenance, inspection, scheduling, and workforce development. The challenge here is implementation. Governance must become part of routine practice rather than remain a formal statement.

A symbiotic-maturity position occurs when the three dimensions develop together. In this position, data and AI systems are reliable and explainable, workers use AI to diagnose problems and improve processes, and governance routines support accountability, consultation, incident review, and continuous learning. The mature position does not mean that AI adoption is complete. It means that the organization has built the capability to keep adapting as models, jobs, skills, and risks evolve.

## 5 Using the HAIAM cube

The HAIAM Cube is intended to support organizations and manufacturing professionals in three practical ways. First, it can support structured reflection on a current configuration. A leadership team, engineering group, union body, or improvement team can map a current AI initiative across the three dimensions. The purpose is not to produce a single score that oversimplifies complexity. The purpose is to identify imbalance. A factory may be relatively advanced in data capability but less advanced in empowerment. Another may be relatively advanced in worker participation but intermediate in governance. The cube makes these imbalances visible and encourages discussion about where improvement should begin, while any formal diagnostic interpretation remains subject to future validation.

Second, the HAIAM Cube can provide a conceptual basis for roadmap discussion. Instead of asking which AI tool should be adopted next, organizations can ask which capability gap prevents responsible progress. If data are fragmented, the next step may be data stewardship rather than another model. If workers do not trust recommendations, the next step may be explainability, consultation, and work redesign. If accountability is unclear, the next step may be an AI governance board with engineering, operations, human resources, safety, and worker representation. In this way, the framework can translate Industry 5.0 values into everyday decisions about budgets, responsibilities, training, and evaluation.

Third, the HAIAM Cube can support professional development for future manufacturing jobs. AI maturity changes what workers need to know, but it should not reduce learning to coding alone. Future manufacturing professionals will need hybrid capabilities. They will need to understand data limitations, interpret AI outputs, communicate with technical specialists, participate in risk assessment, and improve processes collaboratively. This means that augmentation, empowerment, inclusivity, and human-AI symbiosis are not abstract ideals. They are practical job characteristics that can guide training, career pathways, and organizational learning. A technician who can question a maintenance model, explain a false alarm, and help refine a sensor strategy is not merely adapting to AI. The technician is helping the organization become mature.

The HAIAM Cube also has limits. It is conceptual and should be treated as a starting point for empirical validation rather than as a complete measurement instrument. The illustrative indicators, pathways, and maturity positions offered in this article are conceptual propositions rather than validated thresholds, causal mechanisms, or evidence of organizational effects. The article is also a conceptual synthesis based on selected scholarly and policy literature. As no systematic search, selection, quality-appraisal, or synthesis protocol was applied, it should not be interpreted as an exhaustive review of the relevant literature. Future research could develop diagnostic scales for each dimension, test the levels across different manufacturing sectors, and examine whether higher maturity is associated with better outcomes for performance, safety, learning, and worker wellbeing. Case studies could explore how organizations move from one position to another, especially in small and medium-sized manufacturers that may lack extensive data science resources. Comparative research could also examine how regulation, labor institutions, and supply chain relationships shape maturity. Longitudinal and configurational studies are particularly needed to test whether balanced development across the three dimensions predicts augmentation, empowerment, inclusivity, or human-AI symbiosis more strongly than isolated capability gains. These questions matter because Industry 5.0 is not only a technological agenda. It is a practical attempt to align innovation with human capability, social responsibility, and resilience.

## 6 Conclusion

AI adoption in manufacturing is often discussed in terms of tools, use cases, and productivity. These themes are important, but they are not sufficient for Industry 5.0. The deeper question is how organizations can adopt AI in ways that strengthen rather than weaken the future of manufacturing work. The HAIAM Cube responds to this question by conceptually linking three dimensions of maturity to four desired outcomes. AI and data capability provide the technical foundation. Human work capability protects and develops agency. Governance capability ensures that AI is accountable, inclusive, and continuously improved. Together, these dimensions offer propositions about why the same technology can produce very different workplace consequences; they do not yet establish that the proposed maturity configuration causes those consequences.

The proposed HAIAM Cube is intentionally modest. It does not promise a universal route to AI excellence, nor does it assume that maturity is achieved through technology investment alone. Instead, it offers a structured way for organizations to ask better questions. Are models reliable and explainable enough for the work they influence? Are workers empowered to understand and shape AI-supported decisions? Are diverse forms of expertise included in design and evaluation? Are governance routines strong enough to detect risk, learn from failure, and adapt over time? As a conceptual proposition, the cube may help organizations move from fragmented experimentation toward more symbiotic forms of renewal, but this potential contribution requires empirical testing, contextual adaptation, and comparison with alternative frameworks. If future evidence supports the proposed relationships, these questions could become part of everyday manufacturing practice and provide one practical route for advancing the human-centered ambition of Industry 5.0.

## Data Availability

The original contributions presented in the study are included in the article/supplementary material, further inquiries can be directed to the corresponding author.
